# Malnutrition-inflammation-fluid overload complex syndrome and all-cause mortality in patients undergoing hemodialysis

**DOI:** 10.1080/0886022X.2025.2512405

**Published:** 2025-06-04

**Authors:** Rongrong Tian, Liyang Chang, Linghong Cheng, Ruchun Yang, Hongmei Zhang

**Affiliations:** a Department of Nephrology, Hangzhou TCM Hospital Affiliated to Zhejiang Chinese Medical University, Hangzhou, China; b Nephrology Laboratory, Hangzhou TCM Hospital Affiliated to Zhejiang Chinese Medical University, Hangzhou, China

**Keywords:** Extracellular water/total body water ratio, modified creatinine index, high-sensitivity C-reactive protein, hemodialysis, prognosis

## Abstract

**Background:**

Malnutrition, inflammation, and fluid overload can reinforce each other, forming a detrimental syndrome in patients receiving hemodialysis (HD). However, this syndrome remains insufficiently recognized. This study aims to explore the relationship between the malnutrition-inflammation-fluid overload complex syndrome (MIFCS) and all-cause death.

**Methods:**

A retrospective analysis was conducted. Malnutrition, inflammation, and fluid status were evaluated over a 4-month period, while all-cause mortality data were collected during a 7-year follow-up. Fluid status was evaluated using the extracellular water/total body water ratio (ECW/TBW), determined through bioelectrical impedance analysis (BIA). Nutritional status was assessed *via* the modified creatinine index (mCI), while inflammation was assessed through high-sensitivity C-reactive protein (hs-CRP). The Cox proportional hazards model was applied to develop a nomogram model.

**Results:**

A total of 218 patients were included. The simultaneous presence of malnutrition, inflammation, and fluid overload (FO) was linked to the highest mortality risk (HR, 8.908; 95%CI, 2.986–26.575). A nomogram score based on MIFCS was developed to estimate survival probability at 3, 5, and 7 years. An increase in the nomogram score was progressively linked to an elevated mortality risk, with a hazard ratio of 1.399 (95%CI: 1.298–1.508, *p* < 0.001) per 10-point increase.

**Conclusions:**

MIFCS was significantly associated with an elevated mortality risk in HD patients. A comprehensive assessment of malnutrition, inflammation, and FO is essential for accurate prognostic assessment and risk stratification.

## Introduction

Patients undergoing hemodialysis (HD) often suffer from malnutrition and chronic inflammation, both of which can exacerbate the decline of organ function and contribute to the development of atherosclerotic cardiovascular diseases [1,2]. To highlight the connection between malnutrition, inflammation, and cardiovascular complications associated with atherosclerosis, the concepts of ‘malnutrition-inflammation complex syndrome (MICS)’ have been proposed [1,3–5]. However, the concept of MICS might not fully capture the complexity of the issue.

The association between fluid status and adverse clinical outcomes has been established in HD patients [6,7]. However, the parameter is often interpreted in isolation [8]. Indeed, extracellular fluid overload and malnutrition may reinforce each other through inflammation, where fluid overload (FO) itself serves as an inflammatory stimulus [9]. FO could represent part of a broader set of risk factors that negatively affect clinical outcomes. Recently, the concept of the ‘FIFA complex’ has been proposed, representing the syndrome of frailty, inflammation, fluid overload, and atherosclerosis [10]. Frailty can be considered a long-term consequence of malnutrition.

Despite these insights, the combined effects of malnutrition, inflammation, and fluid overload complex syndrome (MIFCS) on clinical outcomes remains insufficiently explored. To bridge this gap, we systematically gathered data on fluid status (extracellular water/total body water), systemic inflammation (high-sensitivity C-reactive protein), and nutritional status. Malnutrition was assessed using the modified creatinine index (mCI), a recently recognized biomarker for frailty, protein-energy wasting (PEW), and sarcopenia [11–14]. The mCI has been shown to be associated with mortality [15,16], and can be easily calculated using variables, including gender, age, single-pool urea Kt/V, and pre-dialysis serum creatinine levels, all of which are conveniently accessible in clinical settings [17]. Our objective is to evaluate the MIFCS by developing a nomogram model and to examine its impact on mortality. Nomograms have proven valuable for predicting disease prognosis and evaluating the combined effect of multiple factors on survival [18–22].

## Materials and methods

### Study settings and participants

This was a retrospective analysis of a longitudinal cohort from our hemodialysis center [23]. Participants were eligible if they were aged 18 years or older and had undergone regular hemodialysis (three sessions per week) for a minimum of three months between March and June 2017. Exclusion criteria included malignancies, acute infections, severe liver disease, limb amputation, wheelchair or bedridden status, pacemaker implantation, hospitalization or cardiovascular events within three months before study initiation. All patients underwent bioimpedance analysis (BIA) tests. The study protocol was approved by hospital’s research ethics committee (No. 2016LSKY). Written informed consent was obtained from all participants.

### Data collection

Demographic, clinical, and biochemical data were gathered from patient records at the time of study recruitment. Cardiovascular history included acute coronary syndrome, cerebrovascular accidents, hospitalizations for congestive heart failure, and peripheral artery disease. All Laboratory analyses, including blood urea nitrogen (BUN), albumin, uric acid (UA), total cholesterol (TCH), serum creatinine (SCr), hemoglobin, calcium, phosphorus, parathyroid hormone (PTH), and ferritin, were performed on fasting blood samples obtained during the study enrollment phase, with standardized collection occurring before the midweek HD session. The adequacy of dialysis was assessed using single-pool Kt/V for urea.

Modified Quantitative Subjective Global Assessment (MQSGA) and mCI were collected to assess nutritional status [17,24]. The mCI was calculated using the formula [17]: Modified creatinine index (mg/kg/d) = 16.21 + 1.12 × [1 if male; 0 if female] − 0.06 × age (years) − 0.08 × single-pool Kt/V for urea + 0.009 × pre-dialysis serum creatinine (μmol/L). Malnutrition was defined as an mCI value ≤ the median cutoff of 21.08 mg/kg/d. Systemic inflammation was assessed using high-sensitivity C-reactive protein (hs-CRP), with inflammation defined as hs-CRP > 6.0 mg/L. Data for total body water (TBW), extracellular water (ECW), intracellular water (ICW) and the ECW/TBW ratio were obtained through BIA conducted after HD treatment. FO was classified as ECW/TBW > 0.390, based on the upper reference limit of the device’s nomogram.

## Outcomes

The primary endpoint was defined as all-cause mortality. All patients were followed until death, renal transplantation, transfer to alternative dialysis facilities, or the completion of the study follow-up [April 2024].

### Statistical analysis

Continuous variables are presented as means ± standard deviations (for normally distributed data) or medians with interquartile ranges (for non-normally distributed data).

The Cox proportional hazards analysis for all-cause mortality was conducted to develop a prognostic model. Variables with *p* < 0.1 in univariate analysis were included in multivariate Cox regression. A nomogram was developed based on independent predictors, assigning each variable a score on a 0–100 scale, with the total score determining survival probability.

To assess the nomogram’s discrimination ability, Harrell’s concordance index (C-index), net reclassification improvement (NRI), and integrated discrimination improvement (IDI) were calculated. The C-index of the base model, incorporating established risk factors, was compared to that of an extended model including mCI, ECW/TBW, and/or hs-CRP. NRI quantified improvements in mortality risk classification, while IDI measured the overall enhancement in predictive accuracy.

Internal validation was conducted using a bootstrap resampling method to ensure an unbiased model performance estimate *via* the C-index. The original cohort underwent 1000 iterations of random sampling with replacement. A calibration curve from bootstrap validation assessed alignment between predicted and observed outcomes.

Statistical analyses were performed by SPSS (version 23.0; IBM, Armonk, NY, USA) and R (version 4.1.3; Vienna, Austria). All tests were two-sided, with a significance level set at *p* < 0.05.

## Results

### Patient characteristics

A total of 218 participants were enrolled between March and June 2017, with a mean age of 61.16 ± 13.74 years, of which 37.6% were women (Table 1). All enrolled patients received high-flux hemodialysis, with a median dialysis vintage of 60.50 months (interquartile range: 33.75–104). The causes of kidney disease included chronic glomerulonephritis (56%), diabetic nephropathy (24.3%), polycystic kidney disease (6.0%), hypertensive nephropathy (4.1%), and other diseases (9.6%). The mean spKt/V was 1.65.

**Table 1.** Clinical characteristics of the participants at the recruitment between March and June 2017.

Characteristics	All (*n* = 218)
Age (years)	61.16 ± 13.74
Sex	
Male, n (%)	136 (62.40%)
Female, n (%)	82 (37.60%)
Dialysis vintage, months	60.50 (33.75, 104.00)
ESRD primary cause	
Diabetic nephropathy, n (%)	53 (24.3%)
Others, n (%)	165 (75.7%)
Comorbid conditions	
History of cardiovascular events, n (%)	27 (12.4%)
Diabetes, n (%)	68 (31.2%)
Smoking, n (%)	30 (13.8%)
Alcohol, n (%)	21 (9.7%)
BMI (kg/m^2^)	21.50 ± 2.81
Kt/V for urea	1.65 ± 0.32
Biological parameters	
BUN (mmol/L)	22.47 ± 5.20
SCr (μmol/L)	896.76 ± 210.38
UA (μmol/L)	442.64 ± 82.06
hs-CRP (mg/L)	2.15 (1.09, 4.28)
Albumin (g/L)	39.24 ± 2.12
TCH (mmol/L)	4.05 ± 0.92
Hemoglobin (g/L)	103.90 ± 13.09
Calcium (mmol/L)	2.32 ± 0.22
Phosphorus (mmol/L)	1.92 ± 0.48
iPTH (pg/mL)	292.85 (156.90, 534.85)
Ferritin (ng/mL)	102.40 (46.98, 226.25)
mCI (mg/kg/d)	21.18 ± 2.50
nPNA (g/kg/d)	1.18 ± 0.27
MQSGA score	12.45 ± 2.98
BIA	
ECW/TBW ratio (%)	38.39 ± 1.06

Values for continuous variables are given as the means ± standard deviations or medians and interquartile ranges. Categorical variables are expressed as numbers (%). BMI, body mass index; BUN, blood urea nitrogen; SCr, serum creatinine; UA, uric acid; hs-CRP, high-sensitivity C-reactive protein; TCH, total cholesterol; iPTH, intact parathyroid hormone; mCI, modified creatinine index; nPNA, normalized protein equivalent of nitrogen appearance; MQSGA, Modified Quantitative Subjective Global Assessment; ECW, extracellular waster; ICW, intracellular water; TBW, total body water.

The patients were followed up for 70 months (interquartile range: 42.75, 85.00). During this period, there were 59 deaths (47 men and 12 women), including 23 deaths due to cardiovascular disease, 12 due to infection, 5 due to malignancies, and 19 due to other causes, respectively.

### Association of malnutrition, inflammation, and FO with survival

In the multivariable-adjusted model, none of the isolated risk conditions (FO, malnutrition, or inflammation alone) were independently associated with all-cause mortality. The coexistence of malnutrition, inflammation, and FO was associated with a higher risk of mortality. The risk of death was greatest when all three factors were present (HR, 8.908; 95%CI, 2.986–26.575) (Table 2). In patients with malnutrition and inflammation, the HR was 4.312 (95%CI: 1.203, 15.457) when ECW/TBW ≤ 0.390; the HR increased to 8.908 (95%CI: 2.986, 26.575) when ECW/TBW > 0.390 (Table 2).

**Table 2.** Associations of the different groups stratified by fluid, malnutrition and inflammation status with mortality.

Fluid status	Malnutrition and inflammation status	n(%)	Death (n)	Unadjusted model		Multivariable-adjusted model^#^	
				Hazard ratio (95% CI)	*p* Value	Hazard ratio (95% CI)	*p* Value
ECW/TBW ≤ 0.390	Without malnutrition and inflammation	89 (40.8)	10	1 (reference)		1 (reference)	
	Only malnutrition or only inflammation	60 (27.5)	14	2.056 (0.913, 4.630)	0.082	1.939 (0.787, 4.776)	0.150
	With malnutrition and inflammation	11 (5)	4	3.610 (1.128, 11.495)	0.031	4.312 (1.203, 15.457)	0.025
ECW/TBW > 0.390	Without malnutrition and inflammation	8 (3.7)	2	2.007 (0.440, 9.162)	0.369	1.605 (0.343, 7.508)	0.548
	Only malnutrition or only inflammation	39 (17.9)	22	6.686 (3.158, 14.154)	< 0.001	3.927 (1.619, 9.526)	0.002
	With malnutrition and inflammation	11 (5)	7	7.396 (2.814, 19.442)	< 0.001	8.908 (2.986, 26.575)	< 0.001

^#^ Adjusted for age, sex, dialysis vintage, history of cardiovascular events, presence of diabetes, history of smoking and alcohol, BMI, Kt/V for urea, normalized protein equivalent of nitrogen appearance, MQSGA score, serum levels of urea nitrogen, uric acid, albumin, total cholesterol, hemoglobin, calcium, phosphorus, parathyroid hormone, and ferritin.

ECW, extracellular waster; TBW, total body water.

### Development of a nomogram model based on MIFCS

The univariate and multivariate Cox proportional hazards analysis results are shown in Table 3. The multivariate analysis showed that, in addition to low mCI (HR, 2.027; 95%CI, 1.041–3.947), hs-CRP (HR, 1.049; 95%CI, 1.027–1.071), and ECW/TBW ratio (HR, 1.681, 95%CI, 1.207–2.341), the following factors were significantly associated with all-cause mortality: age (HR, 1.034; 95%CI, 1.005–1.063), sex (HR, 3.798; 95%CI, 1.945–7.414), and history of cardiovascular events (HR, 2.433; 95%CI, 1.258–4.705) (Table 3).

**Table 3.** Univariable and multivariable Cox analysis for overall survival.

Variables	Univariable*		Multivariable	
	HR (95% CI)	*p* Value	HR (95% CI)	*p* Value
Age (years)	1.068 (1.043, 1.068)	< 0.001	1.034 (1.005, 1.063)	0.021
Sex (male)	3.072 (1.628, 5.797)	0.001	3.798 (1.945, 7.414)	< 0.001
Dialysis vintage (months)	0.996 (0.991, 1.001)	0.085		
History of cardiovascular events	2.173 (1.151, 4.101)	0.017	2.433 (1.258, 4.705)	0.008
Diabetes	2.513 (1.504, 4.199)	< 0.001		
MQSGA	1.092 (1.005, 1.186)	0.038		
Albumin (g/L)	0.840 (0.750, 0.941)	0.003		
UA	0.994 (0.991, 0.998)	0.002		
nPNA	0.378 (0.133, 1.071)	0.067		
mCI (≤ 21.08 vs > 21.08)	3.060 (1.757, 5.916)	< 0.001	2.027 (1.041, 3.947)	0.038
hs-CRP	1.033 (1.015, 1.052)	< 0.001	1.049 (1.027, 1.071)	< 0.001
ECW/TBW ratio (%)	2.302 (1.755, 3.020)	< 0.001	1.681 (1.207, 2.341)	0.002

* Only variables with *p* < 0.1 are shown.

MQSGA, Modified Quantitative Subjective Global Assessment; UA, uric acid; nPNA, normalized protein equivalent of nitrogen appearance; mCI, modified creatinine index; hs-CRP, high-sensitivity C-reactive protein; ECW, extracellular waster; TBW, total body water.

**Figure 1.**
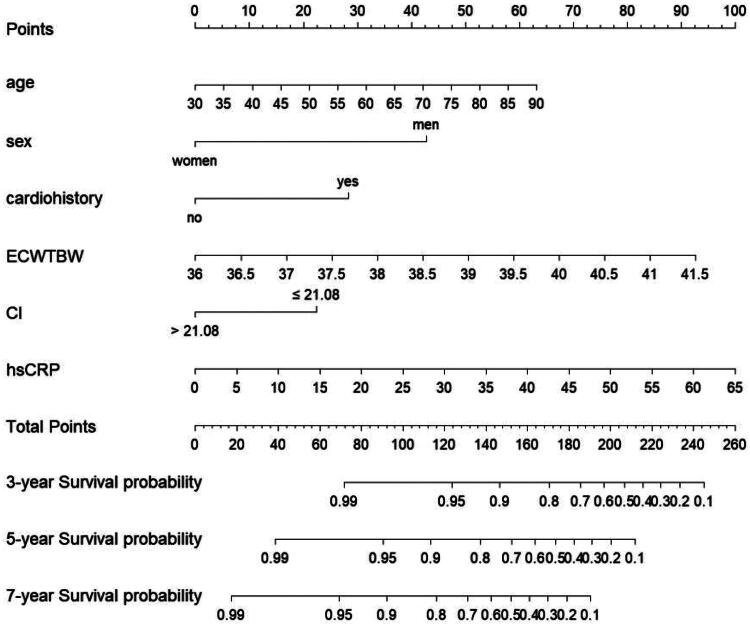
The nomogram for overall survival.ECW, extracellular waster; TBW, total body water; CI, creatinine index; hsCRP, high-sensitivity C-reactive protein.

A nomogram was developed to predict the 3-, 5-, and 7-year survival probabilities for hemodialysis patients by integrating the independent risk factors identified in the multivariate analysis (Figure 1). The nomogram calculates the survival probability by summing the scores of each variable and locating the total score on the scale.

### Discrimination of the nomogram model

**Figure 2.**
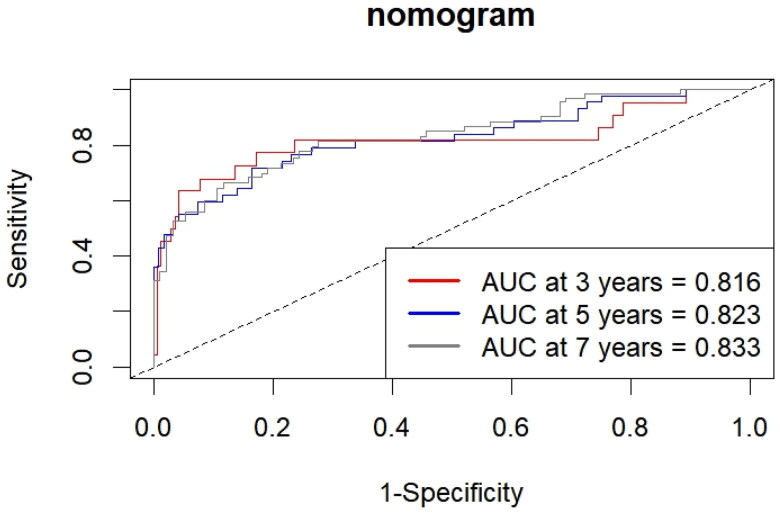
Area under the ROC curves (AUC) for survival prediction.

**Figure 3.**
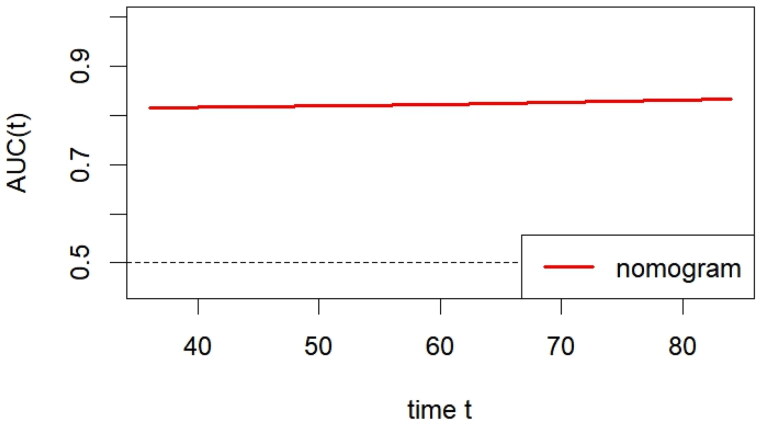
Time-dependent AUC.

The discrimination of the nomogram model was assessed using the C-index, which yielded a value of 0.793 (95% CI: 0.726–0.861). The nomogram demonstrated an AUC of 0.816, 0.823, and 0.833 for predicting 3-, 5-, and 7-year overall survival (OS) (Figure 2). Tim-dependent AUC was also shown (Figure 3).

We compared the discrimination of the Cox models using C statistics (Table 4). The results indicated that the C-index improved when adding mCI alone, ECW/TBW alone, hs-CRP alone, or their combination to the base risk model, which includes age, sex, and history of cardiovascular events. The most significant increase in the C-index occurred when adding the combination of mCI, hs-CRP, and ECW/TBW to the base model (from 0.741 [0.673, 0.809] to 0.793 [0.726, 0.861], *p* < 0.001). This improvement was greater than that observed with the addition of mCI alone (0.767 [0.700, 0.834]), ECW/TBW alone (0.770 [0.705, 0.835]), or hs-CRP alone (0.764 [0.701, 0.828]) to the base model.

**Table 4.** Comparison of C-index of the adjusted cox proportional hazard model.

Models	C-index [95% CI]	*p* Value
Base model**	0.741 [0.673, 0.809]	Ref.
+ mCI	0.767 [0.700, 0.834]	0.001
+ ECWTBW	0.770 [0.705, 0.835]	< 0.001
+ hs-CRP	0.764 [0.701, 0.828]	< 0.001
+mCI, ECWTBW, and hs-CRP	0.793 [0.726, 0.861]^△^^*#^	< 0.001

** The base model includes age, sex, history of cardiovascular events.

^△^ Compared to base model + mCI, *p* < 0.001; *Compared to base model + ECWTBW, *p* < 0.001. ^#^Compared to base model + hs-CRP, *p* < 0.001.

mCI, modified creatinine index; ECW, extracellular waster; TBW, total body water; hs-CRP, high-sensitivity C-reactive protein.

Both NRI and IDI indicated that adding the combination of mCI, hs-CRP, and ECW/TBW to base model improved discrimination of patients at high risk of mortality (NRI, 68.4%, *p* < 0.001; IDI, 10.5%, *p* < 0.001). The discrimination power of the nomogram model was superior to that of the other ­models (Table 5).

**Table 5.** Predictive improvement of the nomogram.

Models	NRI (%) [95%CI]	*p* Value	IDI (%) [95%CI]	*p* Value
Nomogram vs base model**	68.4 [40.1, 96.7]	< 0.001	10.5 [5.4, 15.7]	< 0.001
Nomogram vs (base model + mCI)	40.4 [11.3, 69.6]	0.007	7.2 [2.9, 11.5]	0.001
Nomogram vs (base model + ECW/TBW)	36.7 [7.4, 65.9]	0.014	4.5 [0.9, 8.2]	0.016
Nomogram vs (base model + hs-CRP)	47.1 [18.2, 75.9]	0.001	5.9 [2.3, 9.5]	0.001

** The base model includes age, sex, history of cardiovascular events.

mCI, modified creatinine index; ECW, extracellular waster; TBW, total body water; hs-CRP, high-sensitivity C-reactive protein.

### Internal validation of the nomogram model

**Figure 4.**
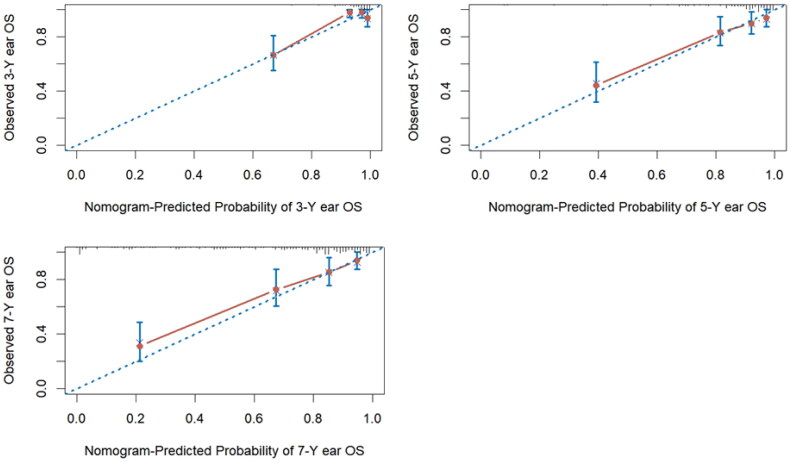
Calibration plots of survival probabilities at 3 years (a), 5 years (B), and 7 years (C). OS, overall survival. Nomogram-predicted overall survival is plotted on the x-axis, with actual overall survival on the y-axis. Dashed lines along the diagonal line through the origin point represent the perfect calibration models in which the predicted probabilities are identical to the observed probabilities.

The nomogram model underwent further validation through internal bootstrap resampling. The C-index was calculated using 1000 bootstrap repetitions, yielding a value of 0.795 (0.724, 0.859), similar to the C-index of the original nomogram. The calibration curve from the internal bootstrap validation demonstrated that the predicted probabilities closely aligned with the observed outcomes, indicating a strong fit and good calibration with the ideal curve (Figure 4).

### Risk stratification based on the nomogram model

The total points could be obtained by summing the scores for each variable in the nomogram model. The risk of all-cause mortality demonstrated a significant and incremental increase with higher nomogram scores (*P* for trend < 0.001, Table 6). When the nomogram score was treated as a continuous variable, similar findings were observed. The hazard ratio for a 10-point increase in the total score was 1.399 (95%CI: 1.298–1.508, *p* < 0.001) (Table 6).

**Table 6.** Association between the nomogram score* and all-cause mortality.

	Unadjusted model			Multivariable-adjusted model^#^		
	Hazard ratio (95% CI)	*p* Value	*P* for trend	Hazard ratio (95% CI)	*p* Value	*P* for trend
Quartiles of nomogram score						
Q1; 19.50–93.64	1 (reference)		*P* < 0.001	1 (reference)		*P* < 0.001
Q2; 93.65–114.94	2.271 (0.603, 8.563)	0.226		1.956 (0.515, 7.429)	0.325	
Q3; 114.95–153.45	4.387 (1.260, 15.275)	0.020		4.719 (1.332, 16.723)	0.016	
Q4. 153.45–247.55	17.044 (5.221, 55.467)	< 0.001		15.661 (4.521, 54.255)	< 0.001	
Every 10 score increase in nomogram score	1.366 (1.271–1.467)	*P* < 0.001		1.399 (1.298, 1.508)	*P* < 0.001	

* The nomogram score is obtained by summing the scores for each variable in the nomogram model.

^#^ Adjusted for dialysis vintage, presence of diabetes, history of smoking and alcohol, BMI, Kt/V for urea, normalized protein equivalent of nitrogen appearance, MQSGA score, serum levels of urea nitrogen, uric acid, albumin, total cholesterol, hemoglobin, calcium, phosphorus, parathyroid hormone, and ferritin.

## Discussion

The findings of this study highlight that the concurrent presence of inflammation, malnutrition, and FO is significantly associated with an increased risk of death. Integrating these factors into a prognostic model enhances the predictive accuracy of outcomes in HD patients.

A key characteristic of malnutrition is sarcopenia and PEW, both of which involve the loss of skeletal muscle mass [25,26]. They are associated with poor outcomes [27,28]. The mCI serves as a marker for muscle metabolism, reflecting muscle mass and function [17]. It has recently been identified as an effective tool for early identification of frailty, sarcopenia, and PEW [11–14]. The results of this study confirm the association between mCI and all-cause mortality, which is in line with recent research [29]. These findings support the use of mCI as a marker of nutritional status and a predictor of clinical outcomes in these patients.

Elevated plasma CRP is recognized as a marker of cytokine-driven acute-phase inflammation. In uremic patients, the inflammatory response may persist due to impaired clearance of pro-inflammatory cytokines and heightened oxidative stress [30]. Our study confirmed that elevated plasma CRP is an independent prognostic factor.

Managing fluid balance is a crucial aspect of dialysis treatment. Fluid overload (FO) is prevalent among HD patients and is strongly linked to cardiovascular events and overall mortality [31,32]. BIA is considered as an optimal method for evaluating fluid status, with the ECW/TBW ratio being a reliable marker of fluid status [8,33]. Our results demonstrated that the fluid status, as reflected by ECW/TBW, is associated with poor outcomes. It has been reported that ECW/TBW is even superior to the cardiac marker N-T-proBNP in predicting mortality [34]. A 16-month cohort study involving peritoneal dialysis and hemodialysis patients showed that the ECW/TBW ratio is a predictor of cardiovascular mortality, irrespective of the type of dialysis modality used [35]. A recent study indicated that post-dialysis ECW/TBW appears to perform better than pre-dialysis ECW/TBW in predicting long-term survival, possibly due to uncorrected fluid overload after dialysis treatment, post-dialysis ECW/TBW better reflects long-term overhydration [8]. Fluid overload significantly impacts vascular and endothelial health, leading to arterial stiffness, atherosclerosis, and left ventricular hypertrophy (LVH), all of which can increase the risk of death [36].

Although fluid overload (FO), inflammation, and malnutrition are independent predictors of mortality, they may complement each other with information, and their combination may contribute to a cumulative risk effect. Current research has validated that the concurrence of any two of these factors significantly increases the risk of mortality. The Malnutrition-Inflammation Score (MIS), which evaluates the combination of malnutrition and inflammation, has been proven to be a major cause of high mortality in MHD patients [37]. And, it was reported that MIS is superior to traditional SGA and MQSGA, as well as to individual laboratory values, as a predictor of clinical outcomes [3]. Additionally, it was reported that the concurrent presence of inflammation and fluid overload increases the risk of mortality in a dose-dependent manner. In the absence of inflammation, the risk of mortality for patients with severe FO is 3.09 times higher than for those without FO; when inflammation is present, this risk increases to 6.02 times [38]. By analyzing changes in fluid and inflammatory status and their relationship with mortality, it was found that patients with persistent inflammation and FO had the highest risk of mortality (HR, 9.44) compared to those who had never experienced FO or inflammation [38].

Few studies have investigated the synergistic effects of fluid overload, inflammation, and malnutrition on clinical outcomes. Our research introduces a nomogram method for evaluating their combined predictive effects. The results demonstrated that incorporating fluid status, malnutrition, and inflammation into the baseline risk model significantly enhanced the accuracy of prognosis prediction. Higher nomogram scores reflect more severe malnutrition, inflammation, and fluid overload, leading to a significantly increased risk of death.

From a pathophysiological perspective, volume status, inflammation, and protein-energy malnutrition can reinforce each other and increased risk of death. For instance, gradual weight loss may go unnoticed, leading to inaccurate dry weight prescription. Additionally, malnutrition increases the risk of hemodynamic instability, making it more difficult to achieve the target dry weight. Furthermore, hypoalbuminemia, associated with malnutrition and inflammation, can lead to fluid redistribution into the interstitial space, which may hinder effective fluid removal during HD [39,40].

The strength of this study lies in introducing the concept of MIFCS, highlighting the importance of a comprehensive assessment of malnutrition, FO, and inflammation while offering a simple and effective tool for survival prediction. However, some limitations should be noted. Data on residual renal function, which could be a key modulating factor for inflammation, malnutrition, and fluid status, were not available in the study. Nonetheless, we believe this is unlikely to significantly impact the main results, as the cohort had a relatively long dialysis vintage, making it improbable that a large portion of patients have residual renal function. In addition, this is a single-center, retrospective study with a relatively small sample size. Although we have performed internal validation using bootstrap resampling, larger, multicenter studies are needed to further validate and confirm these findings.

It needs to be clarified that despite the high predictive value of the nomogram including volume status, malnutriton and inflammation in this study, it cannot be utilized to evaluate its practical usefulness in clinical settings beyond its predictive capability. For example, there is no clear reference cutoff point for measuring overhydration using the ECW/TBW ratio currently, especially in patients with inflammation and malnutrition, where it remains unclear whether attempting to achieve so-called normal hydration status will lead to better outcomes or expose patients to a higher risk of dialysis-related hypotension. Additionally, some studies have observed that post-dialysis fluid depletion is associated with significantly better survival compared to post-dialysis normovolemia [38]. Further clinical studies are needed to clarify these issues.

## Conclusions

MIFCS was strongly associated with an increased risk of all-cause mortality in HD patients. The combined evaluation of malnutrition, inflammation, and fluid status enhances the predictability for all-cause mortality. This approach may help stratify patients more effectively and improve the accuracy of mortality risk prediction.

## Data Availability

The datasets analyzed during the current study are available from the corresponding author on reasonable request.
